# 2585. Identification of Risk Factors for *Pseudomonas aeruginosa* in Patients with Community-Acquired Pneumonia

**DOI:** 10.1093/ofid/ofad500.2200

**Published:** 2023-11-27

**Authors:** Jenna Karajeh, Julia Sapozhnikov, Jenna Adams, Fritzie S Albarillo, Maressa Santarossa, Brian M Hoff

**Affiliations:** Loyola University Medical Center, Oak Forest, Illinois; Loyola University Medical Center, Oak Forest, Illinois; Loyola University Medical Center, Oak Forest, Illinois; Loyola University Medical Center, Oak Forest, Illinois; Loyola University Medical Center, Oak Forest, Illinois; Loyola University Medical Center, Oak Forest, Illinois

## Abstract

**Background:**

Community-acquired pneumonia (CAP) is a leading infectious cause of hospitalization, morbidity, and mortality worldwide. As the etiology of CAP continues to evolve, antibiotic-resistant bacteria are becoming more prevalent in the community setting. Among these pathogens is *Pseudomonas aeruginosa*. Although incidence of CAP due to *P. aeruginosa* is low, it has been linked to severe illness and poor clinical outcomes, highlighting the challenge in treating this infection. Identifying local risk factors for pneumonia due to drug-resistant pathogens, specifically *P. aeruginosa*, can potentially reduce the overuse of unnecessary empiric broad-spectrum antipseudomonal agents and increase appropriateness of empiric antibiotic treatment regimens.

**Methods:**

A single-center, retrospective cohort study was conducted in patients who were treated for CAP at a large academic medical center from August 1, 2019 through July 31, 2022. Specimen data were reviewed and separated into two groups. The *P. aeruginosa* group was composed of *P. aeruginosa*-positive respiratory specimens whereas the non-*P. aeruginosa* group comprised all other pathogens and specimen-negative results. The primary endpoint was to identify risk factors for *P. aeruginosa* in patients with CAP at our institution.

**Results:**

A total of 206 patients were screened and 79 of them were included in the analysis (39 *P. aeruginosa* encounters and 40 non-*P. aeruginosa* encounters). Baseline characteristics were well-distributed between groups. A univariate analysis determined 13 variables were significant at P < 0.2. This P-value was used for entry into the logistic regression model. Of the 13 variables, four were determined to be correlated most closely with CAP due to *P. aeruginosa*: chronic pulmonary disease (OR, 12.4 [95% CI, 2.5 to 61]), prior intravenous antibiotics within the last 12 months (OR, 15.7 [95% CI, 3.2 to 77.2]), sputum production (OR, 8.1 [95% CI, 1.6 to 41.3]), and tube feeds at time of admission (OR, 21.2 [95% CI, 1.2 to 360.5]).

**Conclusion:**

Although the study sample size was small, chronic pulmonary disease, prior intravenous antibiotics within the last 12 months, sputum production, and tube feeds at time of admission correlated most closely with CAP due to *P. aeruginosa* at our institution.

**Disclosures:**

**All Authors**: No reported disclosures

